# Speech Sound Disorders in Children: An Articulatory Phonology Perspective

**DOI:** 10.3389/fpsyg.2019.02998

**Published:** 2020-01-28

**Authors:** Aravind Kumar Namasivayam, Deirdre Coleman, Aisling O’Dwyer, Pascal van Lieshout

**Affiliations:** ^1^Oral Dynamics Laboratory, Department of Speech-Language Pathology, University of Toronto, Toronto, ON, Canada; ^2^Toronto Rehabilitation Institute, University Health Network, Toronto, ON, Canada; ^3^Independent Researcher, Surrey, BC, Canada; ^4^St. James’s Hospital, Dublin, Ireland; ^5^Rehabilitation Sciences Institute, University of Toronto, Toronto, ON, Canada

**Keywords:** speech sound disorders (SSD), Dynamical Systems Theory, Articulatory Phonology, childhood apraxia of speech (CAS), Dysarthria, articulation and phonological disorders, speech motor control, motor speech development

## Abstract

Speech Sound Disorders (SSDs) is a generic term used to describe a range of difficulties producing speech sounds in children (McLeod and Baker, 2017). The foundations of clinical assessment, classification and intervention for children with SSD have been heavily influenced by psycholinguistic theory and procedures, which largely posit a firm boundary between phonological processes and phonetics/articulation (Shriberg, 2010). Thus, in many current SSD classification systems the complex relationships between the etiology (distal), processing deficits (proximal) and the behavioral levels (speech symptoms) is under-specified (Terband et al., 2019a). It is critical to understand the complex interactions between these levels as they have implications for differential diagnosis and treatment planning (Terband et al., 2019a). There have been some theoretical attempts made towards understanding these interactions (e.g., McAllister Byun and Tessier, 2016) and characterizing speech patterns in children either solely as the product of speech motor performance limitations or purely as a consequence of phonological/grammatical competence has been challenged (Inkelas and Rose, 2007; McAllister Byun, 2012). In the present paper, we intend to reconcile the phonetic-phonology dichotomy and discuss the interconnectedness between these levels and the nature of SSDs using an alternative perspective based on the notion of an articulatory “gesture” within the broader concepts of the Articulatory Phonology model (AP; Browman and Goldstein, 1992). The articulatory “gesture” serves as a unit of phonological contrast and characterization of the resulting articulatory movements (Browman and Goldstein, 1992; van Lieshout and Goldstein, 2008). We present evidence supporting the notion of articulatory gestures at the level of speech production and as reflected in control processes in the brain and discuss how an articulatory “gesture”-based approach can account for articulatory behaviors in typical and disordered speech production (van Lieshout, 2004; Pouplier and van Lieshout, 2016). Specifically, we discuss how the AP model can provide an explanatory framework for understanding SSDs in children. Although other theories may be able to provide alternate explanations for some of the issues we will discuss, the AP framework in our view generates a unique scope that covers linguistic (phonology) and motor processes in a unified manner.

## Introduction

In clinical speech-language pathology (S-LP), the distinction between articulation and phonology and whether a speech sound error^1^ arises from motor-based articulation issues or language/grammar based phonological issues has been debated for decades (see Shriberg, 2010; Dodd, 2014; Terband et al., 2019a for a comprehensive overview on this topic). The theory-neutral term Speech Sound Disorders (SSDs) is currently used as a compromise to bypass the constraints associated with the articulation versus phonological disorder dichotomy (Shriberg, 2010). The present definition describes SSD as a range of difficulties producing speech sounds in children that can be due to a variety of limitations related to perceptual, speech motor, or linguistic processes (or a combination) of known (e.g., Down syndrome, cleft lip and palate) and unknown origin (Shriberg et al., 2010; McLeod and Baker, 2017).

The history of causality research for childhood SSDs encompasses several theoretically motivated epochs (Shriberg, 2010). While the first epoch (1920s-1950s) was driven by psychosocial and structuralist views aimed at uncovering distal causes, the second epoch (1960s to 1980s) was driven by psycholinguistic and sociolinguistic approaches and focused on proximal causes. The more recent third and fourth epochs reflect the utilization of advances in neurolinguistics (1990s) and human genome sequencing (post-genomic era; 2000s) and these approaches address both distal and proximal causes (Shriberg, 2010). With these advances, several different systems for the classification of SSD subtypes in children have been proposed based on their distal or proximal cause (e.g., see Waring and Knight, 2013). Some of the major SSD classification systems include the Speech Disorders Classification System (Shriberg et al., 2010), the Model of Differential Diagnosis (Dodd, 2014) and the Stackhouse and Wells (1997) Psycholinguistic Framework. However, a critical problem in these classification systems as noted by Terband et al. (2019a) is that the relationships between the different levels of causation are underspecified. For example, the links between the etiology (distal; e.g., genetics), processing deficits (proximal; e.g., psycholinguistic factors), and the behavioral levels (speech symptoms) are not clearly elucidated. In other words, even though the term SSD is theory-neutral, the poorly specified links between the output level (behavioral) speech symptoms and higher-level motor/language/lexical/grammar processes limits efficient differential diagnosis, customizing intervention and optimizing outcomes (see Terband et al., 2019a for a more detailed review on these issues). Thus, there is a critical need to understand the complex interactions between the different levels that ultimately cause the observable speech symptoms (McAllister Byun and Tessier, 2016; Terband et al., 2019a).

There have been several theoretical attempts at integrating phonetics and phonology in clinical S-LP. In this context, the characterization of speech patterns in children either solely as the product of performance limitations (i.e., challenges in meeting phonetic requirements arising from motor and anatomical differences) or purely as a consequence of phonological/grammatical competence has been challenged (Inkelas and Rose, 2007; Bernhardt et al., 2010; McAllister Byun, 2012). McAllister Byun (2011, 2012) and McAllister Byun and Tessier (2016) suggest a “phonetically grounded phonology” approach where individual-specific production experience and speech-motor development is integrated into the construction of children’s phonological/grammatical representations. The authors discuss this approach using several examples related to the neutralization of speech sounds in word onset (with primary stress) positions. They argue that positional velar fronting in these positions (where coronals sounds are substituted for velar) in children is said to result from a combination of jaw-dominated undifferentiated tongue gesture (e.g., Gibbon and Wood, 2002; see Section “Speech Delay” for details on velar fronting and undifferentiated tongue gestures) and the child’s subtle articulatory efforts (increased linguo-palatal contact into the coronal region) to replicate positional stress (Inkelas and Rose, 2007; McAllister Byun, 2012). McAllister Byun (2012) demonstrated that by encoding this difficulty with a discrete tongue movement as a violable “MOVE-AS-UNIT” constraint, positional velar fronting could be formally discussed within the Harmonic Grammar framework (Legendre et al., 1990). In such a framework the constraint inventory is dynamic and new constraints could be added on the basis of phonetic/speech motor requirements or removed over the course of neuro-motor maturation. In the case of positional velar fronting, the phonetically grounded “MOVE-AS-UNIT” constraint is eliminated from the grammar as the tongue-jaw complex matures (McAllister Byun, 2012; McAllister Byun and Tessier, 2016).

In the present paper, we intend to reconcile the phonetic-phonology dichotomy and discuss the interconnectedness between these levels and the nature of SSDs using an alternative perspective. This alternative perspective is based on the notion of an articulatory “gesture” that serves as a unit of phonological contrast and characterization of the resulting articulatory movements (Browman and Goldstein, 1992; van Lieshout and Goldstein, 2008). We discuss articulatory gestures within the broader concepts of the Articulatory Phonology model (AP; Browman and Goldstein, 1992). We present evidence supporting the notion of articulatory gestures at the level of speech perception, speech production and as reflected in control processes in the brain and discuss how an articulatory “gesture”-based approach can account for articulatory behaviors in typical and disordered speech production (van Lieshout, 2004; van Lieshout et al., 2007; D’Ausilio et al., 2009; Pouplier and van Lieshout, 2016; Chartier et al., 2018). Although, other theoretical approaches (e.g., Inkelas and Rose, 2007; McAllister Byun, 2012; McAllister Byun and Tessier, 2016) are able to provide alternate explanations for some of the issues we will discuss, the AP framework in our view generates a unique scope that covers linguistic (phonology) and motor processes in a unified and transparent manner to generate empirically testable hypotheses. There are other speech production models, but as argued in a recent paper, the majority of those are more similar to the Task Dynamics (TD) framework (Saltzman and Munhall, 1989) in that they address specific issues related to the motor implementation stages (with or without feedback) and not so much include a principled account of phonological principles, such as formulated in AP (Parrell et al., 2019).

## Articulatory Phonology

This section on Articulatory Phonology (AP; Browman and Goldstein, 1992) lays the foundation for understanding speech sound errors in children diagnosed with SSDs from this specific perspective. The origins of the AP model date back to the late 1970s, when researchers at the Haskins laboratories developed a unique and alternative perspective on the nature of action and representation called the Task Dynamics model (TD; Saltzman and Munhall, 1989). This model was inspired by concepts of self-organization related to functional synergies as derived from the Dynamical Systems Theory (DST; Kelso, 1995).

DST in general describes behavior as the emergent product of a “*self organizing, multi-component system that evolves over time*” (Perone and Simmering, 2017, p. 44). Various aspects of DST have been studied and applied in a diverse range of disciplines such as meteorology (e.g., Zeng et al., 1993), oceanography (e.g., Dijkstra, 2005), economics (e.g., Fuchs and Collier, 2007), and medical sciences (e.g., Qu et al., 2014). Recently, there has also been an uptake of DST informed research related to different areas in cognitive and speech-language sciences, including language acquisition and change (Cooper, 1999); language processing (Elman, 1995); development of cognition and action (Thelen and Smith, 1994; Spencer et al., 2011; Wallot and van Orden, 2011); language development (van Geert, 1995, 2008); 2nd language learning and development (de Bot et al., 2007; de Bot, 2008); speech production (see van Lieshout, 2004 for a review; van Lieshout and Neufeld, 2014; van Lieshout, 2017); variability in speech production (van Lieshout and Namasivayam, 2010; Jackson et al., 2016); connection between motor and language development (Parladé and Iverson, 2011); connection between cognitive aspects of phonology and articulatory movements (Tilsen, 2009); and visual word recognition (Rueckle, 2002); and visuospatial cognitive development (Perone and Simmering, 2017).

The role of DST in speech and language sciences, in particular with respect to speech disorders, is still somewhat underdeveloped, mainly because of the challenges related to applying specific DST analyses to the relatively short data series that can be collected in speech research (van Lieshout, 2004). However, we chose to focus on the AP framework, as it directly addresses issues related to phonology and articulation using DST principles related to relative stable patterns of behaviors (attractor states), that emerge when multiple components (neural, muscular, biomechanical) underlying these behaviors interact through time in a given context (self-organization) as shown in the time varying nature of the relationship between coupled structures (synergies) that express those behaviors (Saltzman and Munhall, 1989; Browman and Goldstein, 1992). Some examples of studies using this AP/DST approach can be found in papers on child-specific neutralizations in primary stress word positions (McAllister Byun, 2011), articulation issues related to /r/ production (van Lieshout et al., 2008), apraxia of speech (van Lieshout et al., 2007), studies on motor speech processes involved in stuttering (Saltzman, 1991; van Lieshout et al., 2004; Jackson et al., 2016), phonological development (Rvachew and Bernhardt, 2010), SSDs (Gildersleeve-Neumann and Goldstein, 2015), and in children with repaired cleft-lip histories (van Lieshout et al., 2002). In the next few sections we will review the concept of synergies and the development of speech motor synergies, which are directly related to DST principles of self-organization and coupling, followed by how the AP model uses these concepts to discuss linguistic/phonological contrast.

### Speech Motor Synergies

The concept of speech motor synergy was derived from DST principles based on the notion that complex systems contain multiple (sub)components that are (functionally and/or physically) coupled (Kelso, 1995). This means that these (sub)components interact and function as a coordinated unit where patterns emerge and dissolve spontaneously based on self-organization, that is, without the need of a pre-specified motor plan (Turvey, 1990). These patterns are generated due to internal and external influences relating to inter-relationships between the (sub)components themselves, and the constraints and opportunities for action provided in the environment (Smith and Thelen, 2003). Constraints or specific boundary conditions that influence pattern emergence may relate to physical, physiological, and functional/task constraints (e.g., Diedrich and Warren, 1995; Kelso, 1995; van Lieshout and Namasivayam, 2010). Such principles of pattern formation and coupling have already been demonstrated in physical (e.g., Gunzig et al., 2000) and biological systems (e.g., Haken, 1985), including neural network dynamics (e.g., Cessac and Samuelides, 2007). Haken et al. (1985), Kelso et al. (1985), and Turvey (1990) at the time were among the first to apply these principles also to movement coordination. Specifically, a *synergy* in the context of movement is defined as a functional assembly of (sub)components (e.g., neurons, muscles, joints) that are temporarily coupled or assembled in a task-specific manner, thus constrained to act as a single coordinated unit (or a coordinative structure; Kelso, 1995; Kelso et al., 2009). In motor control literature, the concept of coordinative structures or functional synergies are typically modeled as (non-linear) oscillatory systems (Kelso, 1995; Newell et al., 2003; Profeta and Turvey, 2018). By strengthening or weakening the coupling within and between the system’s interacting (sub)components, synergies may be tuned or altered. For movement control, the synergy tuning process occurs with development and learning or may change due to task demands or constraints (e.g., Smith and Thelen, 2003; Kelso et al., 2009).

With regards to speech production, perturbation paradigms similar to the ones used in other motor control studies have demonstrated critical features of oral articulatory synergies (e.g., Folkins and Abbs, 1975; Kelso and Tuller, 1983; van Lieshout and Neufeld, 2014), which in AP terms can be referred to as gestures. Functional synergies in speech production comprise of laryngeal and supra-laryngeal structures (tongue, lips, jaw) coupled to achieve a single constriction (location and degree) goal. Perturbing the movement of one structure will lead to compensatory changes in all functionally coupled structures (including the articulator that is perturbed) to achieve the synergistic goal (Kelso and Tuller, 1983). For example, when the jaw is perturbed in a downward direction during a bilabial stop closure, there is an immediate compensatory lowering of the upper lip and an increased compensatory elevation of the lower lip (Folkins and Abbs, 1975). The changes in the nature and stability of movement coordination patterns (i.e., within and between specific speech motor synergies) as they evolve through time can be captured quantitatively via *order parameters* such as relative phase. Relative phase values are expressed in degrees or radians, and the standard deviation of relative phase values can provide an index of the stability of the couplings (Kelso, 1995; van Lieshout, 2004). Whilst order parameters capture the relationship between the system’s interacting (sub)components, changes in order parameter dynamics can be triggered by alterations in a set of control parameters. For example, changes in movement rate may destabilize an existing coordination pattern and result in a different coordination pattern as observed during gait changes (such as switching from a walk to a trot and then a gallop) as a function of required locomotion speed (Hoyt and Taylor, 1981; Kelso, 1995). For speech, such distinct behavioral patterns as a function of rate have not been established. However, in the coordination between lower jaw, upper and lower lip as part of a lip closing/opening synergy, typical speakers have shown a strong tendency for reduced covariance in the combined movement trajectory, despite individual variation in the actual sequence and timing of individual movements (Alfonso and van Lieshout, 1997). This can be considered a characteristic of an efficient synergy. The same study also included people who stutter and reported more instances of not showing reduced covariance in this group, in line with the notion that stuttering is related to limitations in speech motor skill (van Lieshout et al., 2004; Namasivayam and van Lieshout, 2011).

Recent work has provided more insights regarding cortical networks in control of this coordination between speech articulators (Bouchard et al., 2013; Chartier et al., 2018). Chartier et al. (2018) mapped acoustic and articulatory kinematic trajectories to neural electrode sites in brains of patients, as part of their clinical treatment of epilepsy. Similar to limb control studies that discovered single motor cortical neurons that encoded complex coordinated arm and hand movements (Aflalo and Graziano, 2006; Saleh et al., 2012), coordinated movements involving articulators for specific vocal-tract configurations were encoded at the single electrode level in the ventral sensorimotor cortex (vSMC). That is, activity in the vSMC reflects the synergies used in speech production rather than individual movements. Interestingly, the study found four major clusters of articulatory kinematic trajectories that encode the main vocal tract configurations (labial, coronal, dorsal, and vocalic) necessary to broadly represent the production of American English sounds. The encoded articulatory kinematic trajectories exhibited damped oscillatory dynamics as inferred from articulatory velocity and displacement relationships (phase portraits). These findings support theories that envision vocal tract gestures as articulatory units of speech production characterized by damped oscillatory dynamics [Fowler et al., 1980; Browman and Goldstein, 1989; Saltzman and Munhall, 1989; see Section Articulatory Phonology and Speech Sound Disorders (SSD) in Children].

The notion of gestures at the level of speech perception has been discussed in the Theory of Direct Perception (Fowler, 1986; Fowler and Rosenblum, 1989). This theory posits that listeners perceive attributes of vocal tract gestures, arguing that this reflects the common code shared by both the speaker and listener (Fowler, 1986, 1996, 2014; Fowler and Rosenblum, 1989). These concepts are supported by a line of research studies which propose that the minimal objects of speech perception reflect gestures realized by the action of coordinative structures as transmitted by changes to the acoustic (and visual) signal, rather than units solely defined by a limited set of specific acoustic features (Diehl and Kluender, 1989; Fowler and Rosenblum, 1989; Fowler, 1996). The Direct Perception theory thus suggests that speech perception is driven by the structural global changes in external sensory signals that allow for direct recognition of the original (gesture) source and does not require special speech modules or the need to invoke the speech motor system (Fowler and Galantucci, 2005). Having a common unit for production and perception provides a useful framework to understand the broader nature of both sensory and motor involvement in speech disorders. For example, this can inform future studies to investigate how problems in processing acoustic information and thus perceiving the gestures from the speaker, may interfere with the tuning of gestures for production during development. Similarly, issues related to updating the state of the vocal tract through somato-sensory feedback (a critical component in TD; Saltzman and Munhall, 1989; Parrell et al., 2019) during development may also lead to the mistuning of gestures in production, potentially leading to the type of errors in vocal tract constriction degree and/or location as discussed in Section “Articulatory Phonology and Speech Sound Disorders (SSD) in Children.” However, for the current paper, the focus will be on production aspects only.

### Development of Speech Motor Synergies

**FIGURE 1:**
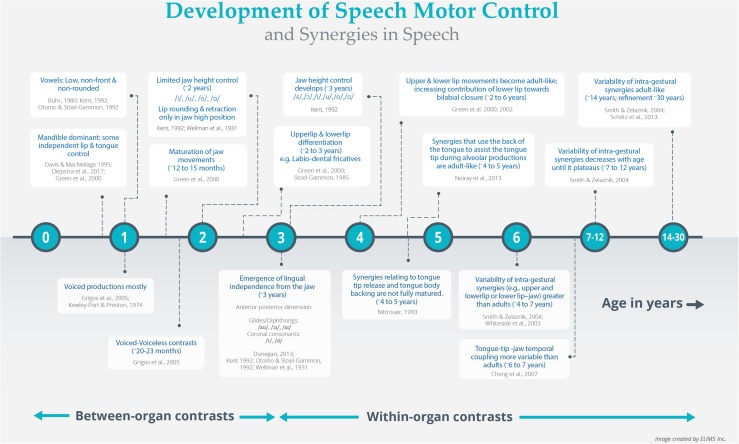
Data driven timeline map of the development of speech motor control and articulatory synergies.

In this section, we will discuss the development and refinement of articulatory synergies and how these processes facilitate the emergence of speech sound contrasts. Observational and empirical data from several speech motor studies (as discussed below) were synthesized to create the timeline map of the development and refinement of speech motor control and articulatory synergies as illustrated in Figure 1. Articulatory synergies in infants have distinct developmental schedules. Speech production in infants is thought to be restricted to sounds primarily supported by the mandible (MacNeilage and Davis, 1990; Davis and MacNeilage, 1995; Green et al., 2000). Early mandibular movements (∼1 year or less) are ballistic in nature and restricted to closing and opening gestures due to the limited fine force control required for varied jaw heights (Locke, 1983; Kent, 1992; Green et al., 2000). Vowel productions in the first year are generally related to low, non-front, and non-rounded vowels; implying that the tongue barely elevates from the jaw, and there is limited facial muscle (lip) interaction (i.e., synergy) with the jaw (Buhr, 1980; Kent, 1992; Otomo and Stoel-Gammon, 1992; but see Giulivi et al., 2011; Diepstra et al., 2017).

Sound sequences that do not require complex timing and coordination within/between articulatory gestures are easier to produce and the first to emerge (Green et al., 2000; Green and Nip, 2010; Figure 1). For instance, young children are unable to coordinate laryngeal voicing gesture with supra-laryngeal articulation and hence master voiced consonants and syllables earlier than voiceless ones (Kewley-Port and Preston, 1974; Grigos et al., 2005). The synergistic interaction between the laryngeal and supra-laryngeal structures underlying voicing contrasts is acquired closer to 2 years of age (∼20–23 months; Grigos et al., 2005), and follows the maturation of jaw movements (around 12–15 months of age; Green et al., 2002; Figure 1) and/or jaw stabilization (Yu et al., 2014).

In children, up to and around 2 years of age, there is limited fine motor control of jaw height (or jaw grading) and weak jaw-lip synergies during bilabial production, but relatively stronger inter-lip spatial and temporal coupling (Green et al., 2000, 2002; Nip et al., 2009; Green and Nip, 2010). A possible consequence of these interactions is that their production of vowels is limited to that of extremes (high or low; /i/, /u/, /o/, and /ɑ/), and lip rounding/retraction is only present when the jaw is in a high position (Wellman et al., 1931; Kent, 1992; Figure 1). As speech-related jaw-lip synergies are emerging, it is not surprising that children’s ability to execute lip rounding and retraction is possible when degrees of freedom can be reduced (i.e., when jaw is held in a high position). Observation of such a reduction in degrees of freedom in emerging synergies has been observed in other non-speech systems (Bernstein, 1996). Interestingly, although the relatively strong inter-lip coordination pattern found in 2-year-olds is facilitative for bilabial productions, it needs to further differentiate to gain independent control of the functionally linked upper and lower lips prior to the emergence of labio-dental fricatives (/f/ and /v/; Green et al., 2000; Figure 1). This process is observed to occur between the ages of 2 and 3 years (Stoel-Gammon, 1985; Green et al., 2000). Green et al. (2000, 2002) suggest that upper and lower lip movements become adult-like with increasing contribution of the lower-lip toward bilabial closure between the ages of 2 and 6 years. Further control over jaw height (with the addition of /ε/ and /ɔ/) and lingual independence from the jaw is developed around 3 years of age (Kent, 1992). The latter is evident from the production of reliable lingual gliding movements (diphthongs: /aʊ/, /ɔɪ/, and /aɪ) in the anterior-posterior dimension (Wellman et al., 1931; Kent, 1992; Otomo and Stoel-Gammon, 1992; Donegan, 2013). Control of this dimension also coincides with the emergence of coronal consonants (e.g., /t/ and /d/; Smit et al., 1990; Goldman and Fristoe, 2000). By 4 years of age, all front and back vowels are within the spoken repertoire of children, suggesting a greater degree of control over jaw height and improved tongue-jaw synergies (Kent, 1992). Intriguingly, front vowels and lingual coronal consonants emerge relatively late (Wellman et al., 1931; Kent, 1992; Otomo and Stoel-Gammon, 1992). This is possibly due to the fine adjustments required by the tongue tip and blade to adapt to mandibular angles. Since velar consonants and back vowels are produced by the tongue dorsum, they are closer to the origin of rotational movement (i.e., condylar axis) and are less affected than the front vowels and coronal consonants (Kent, 1992; Mooshammer et al., 2007). With maturation and experience, finer control over tongue musculature develops, and children begin to acquire rhotacized (retroflexed or bunched tongue) vowels (/ɝ/ and /ɚ/) and tense/lax contrasts (Kent, 1992).

The later development of refined tongue movements is not surprising, since the tongue is considered a hydrostatic organ with distinct functional segments (e.g., tongue tip, tongue body; Green and Wang, 2003; Noiray et al., 2013). Gaining motor control and coordinating the tongue with neighboring articulatory gestures is difficult (Kent, 1992; Smyth, 1992; Nittrouer, 1993). Cheng et al.’s (2007) study demonstrated a lower degree and more variable tongue tip to jaw temporal coupling in 6- to 7-year-old children relative to adults (Figure 1). This contrasts with the earlier developing lip-jaw synergy reported by Green et al. (2000), wherein by 6 years of age, children’s temporal coupling of lip and jaw was similar to adults. The coordination of the tongue’s subcomponents follows different maturation patterns. By 4–5 years, synergies that use the back of the tongue to assist the tongue tip during alveolar productions are adult-like (Noiray et al., 2013), while synergies relating to tongue tip release and tongue body backing are not fully mature (Nittrouer, 1993; Figure 1). The extent and variability of lingual vowel-on-consonant coarticulation between 6 and 9 years of age is greater than in adults; implying that children are still refining their tuning of articulatory gestures (Nittrouer, 1993; Nittrouer et al., 1996, 2005; Cheng et al., 2007; Zharkova et al., 2011).

These findings suggest that articulatory synergies have varying schedules of development: lip-jaw related synergies develop earlier than tongue-jaw or within tongue-related synergies (Cheng et al., 2007; Terband et al., 2009). Most of this work has been done on intra-gestural coordination (i.e., between individual articulators within a gesture), but it is clear that both the development of intra- and inter-gestural synergies are non-uniform and protracted (Whiteside et al., 2003; Smith and Zelaznik, 2004). Variability of intra-gestural synergies (e.g., upper- and lower-lip or lower lip–jaw) in 4- and 7-year-olds has been found to be greater than with adults but decreases with age until it plateaus between 7 and 12 years (Smith and Zelaznik, 2004). Adult-like patterns are reached at around 14 years, and likely continuously refine and stabilize even up to the age of 30 years (Smith and Zelaznik, 2004; Schötz et al., 2013; Figure 1). Overall, these findings suggest that the development of speech motor control is hierarchical, sequential, non-uniform, and protracted.

### Gestures, Synergies and Linguistic Contrast

**FIGURE 2:**
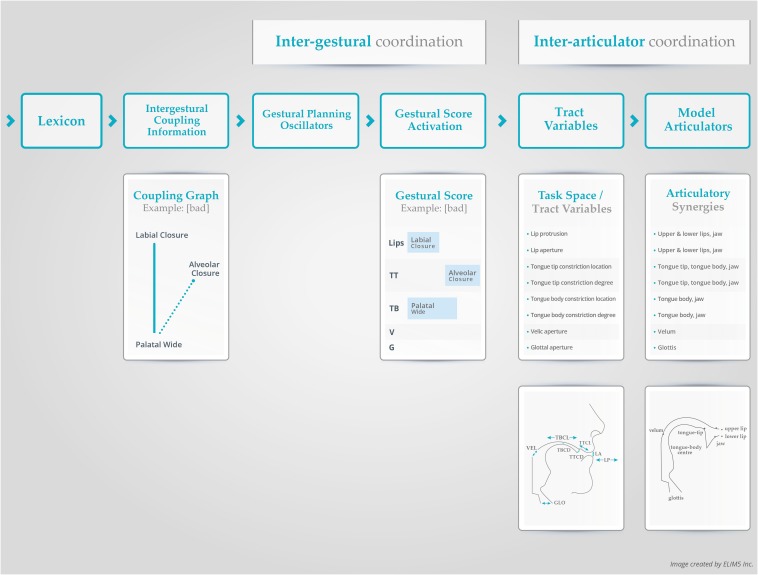
A schematic representation of the AP model with key components (Nam and Saltzman, 2003; Goldstein et al., 2007). TT, tongue tip; TB, tongue body; CD, constriction degree; CL, constriction location; Vel (or V in panel 3), Velum; GLO (or G in panel 3), glottis; LA, lip aperture; LP, lip protrusion (see text for more details).

As mentioned above, within the AP model, the fundamental units of speech are articulatory “gestures.” Articulatory “gestures” are higher-level abstract specifications for the formation and release of task-specific, linguistically relevant vocal tract constrictions. The specific goals of each gesture are defined as *Tract Variables* (Figure 2) and relate to vocal tract constriction location (labial, dental, alveolar, postalveolar, palatal, velar, uvular, and pharyngeal) and constriction degree (closed, critical, narrow, mid, and wide; Figure 2). While constriction degree is akin to manner of production (e.g., fricatives /s/ and /z/ are assigned a “critical” value; stops /p/ and /b/ are given a “closed” value), constriction location allows for distinctions in place of articulation (Browman and Goldstein, 1992; Gafos, 2002).

The targets of each *Tract Variable* are implemented by specifying the lower-level functional synergy of individual articulators (e.g., articulator set of lip closure gesture: upper lip, lower lip, jaw) and their associated muscles ensembles (e.g., orbicularis oris, mentalis, risorius), which allows for the flexibility needed to achieve the task goal (Saltzman and Kelso, 1987; Browman and Goldstein, 1992; Alfonso and van Lieshout, 1997; Gafos, 2002; Figure 2). The coordinated actions of the articulators toward a particular value (target) of a *Tract Variable* is modeled using damped mass spring equations (Saltzman and Munhall, 1989). The variables in the equations specify the final position, the time constant of the constriction formation (i.e., the speed at which the constriction should be formed; stiffness), and a damping factor to prevent articulators from overshooting their targets (Browman and Goldstein, 1989; Kelso et al., 1986a, b; Saltzman and Munhall, 1989). For example, if the goal is to produce constriction at the lips (bilabial closure gesture), then the distance between the upper lip and lower lip (lip aperture) is set to zero. The resulting movements of individual articulators lead to changes in vocal tract geometry, with predictable aerodynamic and acoustic consequences.

The flexibility within the functional articulatory synergy implies that the task-level goals could be achieved with quantitatively different contributions from individual articulatory components as observed in response to articulatory perturbations or in adaptation to the linguistic context in which the gesture is produced (Saltzman and Kelso, 1987; Browman and Goldstein, 1992; Alfonso and van Lieshout, 1997; Gafos, 2002). In other words, the task-level goals are discrete, invariant or context-free, but the resulting articulatory motions are context-dependent (Browman and Goldstein, 1992). Gestures are phonological primitives that are used to achieve linguistic contrasts when combined into larger sequences (e.g., segments, words, phrases). The presence or absence of a gesture, or changes in gestural parameters like constriction location results in phonologically contrastive units. For example, the difference between “bad” and “ban” is the presence of a velum gesture in the latter, while “bad” and “pad” are differentiated by adding a glottal gesture for the onset of “bad”. Parameter differences in gestures such as the degree of vocal tract constriction yields phonological contrast by altering manner of production (e.g., “but” and “bus”; tongue tip constriction degree: complete closure for /t/ vs. a critical opening value to result in turbulence for /s/) (Browman and Goldstein, 1986, 1992; van Lieshout et al., 2008).

Gestures have an internal temporal structure characterized by landmarks (e.g., onset, target, release) which can be aligned to form segments, words, sentences and so on (Gafos, 2002). These gestures and their timing relationships are represented by a gestural score in the AP model (Figure 2; Browman and Goldstein, 1992). Gestural scores are estimated from articulatory kinematic data or speech acoustics by locating kinematic/acoustic landmarks to determine the timing relationships between gestures (Nam et al., 2012). The timing relationships in the gestural score are typically expressed as relative phase values (Kelso et al., 1986a,b; van Lieshout, 2004). Words may differ by altering the relative phasing between their component gestures. For example, although the gestures are identical in “pat” and “tap,” the relative phasing between the gestures are different (Saltzman and Byrd, 2000; Saltzman et al., 2006; Goldstein et al., 2007). As mentioned above, the coordination between individual gestures in a sequence is referred to as inter-gestural coupling/coordination (van Lieshout and Goldstein, 2008). Inter-gestural level timing is not rigidly specified across an entire utterance but is sensitive to peripheral (articulatory) events (Saltzman et al., 1998; Namasivayam et al., 2009; Tilsen, 2009). The presence of a coupling between inter-gestural level timing oscillators and feedback signals arising from the peripheral articulators was identified in experimental work by Saltzman et al. (1998). In that study, unanticipated lip perturbation during discrete and repetitive production of the syllable /pa/ resulted in phase-shifts in the relative timing between the two independent gestures (lip closure and laryngeal closure) for the phoneme /p/ and between successive /pa/ syllables (Saltzman et al., 1998). This confirms the critical role of somato-sensory information in the TD model (Saltzman and Munhall, 1989; Parrell et al., 2019).

Dynamical systems can express different self-organizing coordination patterns, but for many systems, certain patterns of coordination seem to be preferred over others. These preferred patterns are induced by “attractors” (Kelso, 1995), which reflect stable states in the coupling dynamics of such a system^2^. The coupling relationships used in speech production are similar to those identified for limb control systems (Kelso, 1995; Goldstein et al., 2006) and capitalize on intrinsically stable modes of coordination (specifically, in-phase and anti-phase modes; Haken et al., 1985). These are patterns that are naturally achieved without training or learning; however, they are not equally stable (Haken et al., 1985; Nam et al., 2009). In-phase coordination patterns, for instance, are relatively more stable than anti-phase patterns (Haken et al., 1985; Kelso, 1995; Goldstein et al., 2006). Other coordination patterns are possible, but they are more variable, may require higher energy expenditure and can only be acquired with significant training (Kelso, 1984; Peper et al., 1995; Peper and Beek, 1998; Nam et al., 2009). For example, when participants are asked to oscillate two limbs or fingers, they spontaneously switch coordination patterns from the less stable anti-phase to the more stable in-phase as the required movement frequency increases, but not vice versa (Kelso, 1984; Haken et al., 1985; Peper et al., 2004). These two modes of coordination likely form the basis of syllable structure (Goldstein et al., 2006). The onset consonant (C) and vowel (V) planning oscillators (see below) are said to be coupled in-phase, while the CC onset clusters and the nucleus (V) and coda (C) gestures are coupled in anti-phase mode. As the in-phase coupling mode is more stable, this can explain the dominance of CV syllable structure during babbling and speech development as well as across languages (Goldstein et al., 2006; Nam et al., 2009; Giulivi et al., 2011).

Using the TD framework in the AP model (Nam and Saltzman, 2003), speech production planning processes and dynamic multi-frequency coupling between gestural and rhythmic (prosodic) systems have been explained using the notion of coupled oscillator models (Goldstein et al., 2006; Nam et al., 2009; Tilsen, 2009; Gafos and Goldstein, 2012). The coupled oscillator models for speech gestures are associated with non-linear (limit cycle) planning level oscillators which can be coordinated in relative time by specifying a phase relationship between them. During an utterance, the planning oscillators for multiple gestures generate a representation of the various (and potentially competing) coupling specifications, referred to as a coupling graph (Figure 2; Saltzman et al., 2006). The activation of each gesture is then triggered by its respective oscillator after they settle into a stable pattern of relative phasing during the planning process (van Lieshout and Goldstein, 2008; Nam et al., 2009). In this manner, the coupled oscillator model has been used to control the relative timing of multiple gestural activations during word or sentence production. To recap, individual gestures are modeled as critically damped mass-spring systems with a fixed-point attractor where speed, amplitude and duration are manipulated by adjustments to dynamic parameter specifications (e.g., damping and stiffness variables). In contrast, gestural planning level systems are modeled using limit cycle oscillators and their relative phases are controlled by potential functions (Tilsen, 2009; Pouplier and Goldstein, 2010).

Similar to the bidirectional relationship between inter-gestural timing and peripheral articulatory state, interactions between gestural and rhythmic level oscillators have also been noted. To explain the dynamic interactions between gestural and rhythmic (stress and prosody) systems, speech production may rely on a similar multi-frequency system of coupled oscillators as proposed for limb movements (Peper et al., 1995; Tilsen, 2009). The coupling strength and stability in such systems varies not only as a function of type of phasing (in-phase or anti-phase), but also by the complexity of coupling (ratio of intrinsic oscillator frequencies of the coupled structures), movement amplitude and the movement rate at which the coupling needs to be maintained (Peper et al., 1995; Peper and Beek, 1998; van Lieshout and Goldstein, 2008; van Lieshout, 2017). For example, rhythmic movement between the limbs has been modeled as a system of coupled oscillators that exhibit (multi)frequency locking. The most stable coupling mode is when two or more structures (oscillators) are frequency locked in a lower-order (e.g., 1:1) ratio. Multi-frequency locking for upper limbs is possible at higher order ratios of 3:5 or 5:2 (e.g., during complex drumming) but only at slower movement frequencies. As the required movement rate increases, the complex frequency coupling ratios will exhibit transitions to simpler and inherently more stable ratios (Peper et al., 1995; Haken et al., 1996). Studies on rhythmic limb coupling show that increases in movement frequency are inversely related to decreases in coupling strength and coordination stability. The increases in movement frequency or rate may be associated with a drop in the movement amplitude that mediates the differential loss of stability across the frequency ratios (Haken et al., 1996; Goldstein et al., 2007; van Lieshout, 2017). However, smaller movement amplitude in itself (independent from duration and rate) can also decrease coupling strength and coordination stability (Haken et al., 1985; Peper et al., 2008; van Lieshout, 2017). Amplitude changes are presumably used to stabilize the output of a coupled neural oscillatory system. Smaller movement amplitudes may decrease feedback gain, resulting in a reduction of the neural oscillator-effector coupling strength and stability (Peper and Beek, 1998; Williamson, 1998; van Lieshout et al., 2004; van Lieshout, 2017). Larger movement amplitudes facilitate neural phase entrainment by enhancing feedback signals, but a certain minimum sensory input is required for entrainment to occur (Williamson, 1998; Ridderikhoff et al., 2005; Peper et al., 2008; Kandel, 2013; van Lieshout, 2017). Several studies have demonstrated the critical role of movement amplitude on coordination stability in different types of speech disorders such as stuttering and apraxia (van Lieshout et al., 2007; Namasivayam et al., 2009; for review see Namasivayam and van Lieshout, 2011).

Such complex couplings between multi-frequency oscillators may be found at different levels in the speech system such as between slower vowel production and faster consonantal movements (Goldstein et al., 2007), or between shorter-time scale gestures and longer-time scale rhythmic units (moras, syllables, feet and phonological phrases; Tilsen, 2009). Experimentally, the interaction between gestural and rhythmic systems have been identified by a high correlation between inter-gestural temporal variability and rhythmic variability (Tilsen, 2009), while behaviorally, such gesture-rhythm interactions are supported by observations of systematic relationships between patterns of segment and syllable deletions, and stress patterns in a language (Kehoe, 2001; for an alternative take on neutralization in strong positions using constraint-based theory and AP model see McAllister Byun, 2011). Issues in maintaining the stability of complex higher order ratios in multi-frequency couplings (especially at faster speech rates) between slower vowel production and faster consonantal movements have also been implicated in the occurrence of speech sound errors in healthy adult speakers (Goldstein et al., 2007). More about this aspect in the next section.

The development of gestures is tied to organs of constriction in two ways: between-organ and within-organ differentiation (Goldstein and Fowler, 2003). There is empirical data to support that these differentiations occur over developmental timelines (Cheng et al., 2007; Terband et al., 2009; see Section Development of Speech Motor Synergies). When a gesture corresponds to different organs (e.g., bilabial closure implemented via upper and lower lip plus jaw), between-organ differentiation is observed at an earlier stage in development. For within-organ differentiation, children must learn that for a given organ, different gestures may require different variations in vocal tract constriction location and degree. For example, /d/ and /k/ are produced by the same constriction organ (tongue) but use different constriction locations (alveolar vs. velar). Within-organ differentiation is said to occur at a later stage in development via a process called attunement (Studdert-Kennedy and Goldstein, 2003). During the attunement process, initial speech gestures produced by an infant (i.e., based on between organ contrasts) become tailored (attuned) toward the perceived finer grained differentiations in gestural patterns in the ambient language (e.g., similar to phonological attunement proposed by Shriberg et al., 2005). In sum, gestural planning, temporal organization of gestures, parameter specification of gestures, and gestural coupling (between gestures, and between gestures and other rhythmic units) result in specific behavioral phenomena including casual speech alternations (e.g., syllable deletions, assimilations), as will be discussed next.

### Describing Casual Speech Alternations

The AP model accounts for variations and errors in the speech output by demonstrating how the task-specific gestures at the macroscopic level are related to the systematic changes at the microscopic level of articulatory trajectories and resulting speech acoustics (e.g., speech variability, coarticulation, allophonic variation, and speech errors in casual connected speech; Saltzman and Munhall, 1989; Browman and Goldstein, 1992; Goldstein et al., 2007). Browman and Goldstein (1990b) argue that speech sound errors such as consonant deletions, assimilations, and schwa deletions can result from an increasing overlap between different gestures, or from reducing the size (magnitude) of articulatory gestures (see also van Lieshout and Goldstein, 2008; Hall, 2010). The amount of gestural overlap is assumed to be a function of different factors, including style (casual vs. formal speech), the organs used for making the constrictions, speech rate, and linguistic constraints (Goldstein and Fowler, 2003; van Lieshout and Goldstein, 2008).

The gestural processes surrounding consonant and schwa deletions can be explained by alterations in gestural overlap resulting from changes in relative timing or phasing in the gestural score. The gestural overlap has different consequences in the articulatory and acoustic output, depending on whether the gestures share the same *Tract Variables* and corresponding articulatory sets (homorganic) or whether they employ different *Tract Variables* and constricting organs (heterorganic). Heterorganic gestures (e.g., lip closure combined with a tongue tip closure) will result in a *Tract Variable* motion for each gesture that is unaffected by the other concurrent gesture; and their *Tract Variables* goals will be reached, regardless of the degree of overlap. However, when maximum overlap occurs, one gesture may completely obscure or hide the other gesture acoustically during release (i.e., gestural hiding; Browman and Goldstein, 1990b). In homorganic gestures, when two gestures share the same *Tract Variables* and articulators, as in the case of a tongue tip (TT) constriction to produce /θ/ and /n/ (e.g., during production of /tεn θimz/) they perturb each other’s *Tract Variable* motions. The dynamical parameters of the two overlapping gestural control regimes are ‘blended.’ These gestural blendings are traditionally described phonologically as *assimilation* (e.g., /tεn θimz/ → [tεn̪ θimz]) or allophonic variations (e.g., front and back variation of /k/ in English: “*key*” and “*caw*”; Ladefoged, 1982) (Browman and Goldstein, 1990a,b).

Articulatory kinematic data collected using an X-Ray Microbeam system (e.g., Browman and Goldstein, 1990b) have provided support for the occurrence of these gestural processes (hiding and blending). Consider the following classic examples in the literature (Browman and Goldstein, 1990b). The production of the sequence *“nabbed most”* is usually heard by the listener as *“nab most”* and the spectrographic display reveals no visible presence of /d/. However, the presence of the tongue tip raising gesture for /d/ can be seen in X-ray data (Browman and Goldstein, 1990b), but it is inaudible and completely overlapped by the release of the bilabial gestures /b/ and /m/ (Hall, 2010). Similarly, in fast speech, words like “*potential”* sound like *“ptential,”* wherein the first schwa between the consonants /p/ and /t/ seems to be omitted, but in fact is hidden by the acoustic release of /p/ and /t/ (Byrd and Tan, 1996; Davidson, 2006; Hall, 2010). These cases show that relevant constrictions are formed, but they are acoustically and perceptually hidden by another overlapping gesture (Browman and Goldstein, 1990b). Assimilations have also been explained by gestural overlap and gesture magnitude reduction. In the production of “*seven plus seven*,” which often sounds like “*sevem plus seven*,” the coronal nasal consonant /n/ appears to be replaced by the bilabial nasal /m/ in the presence of the adjacent bilabial /p/. In reality, the tongue tip /n/ gesture is reduced in magnitude and overlapped by the following bilabial gesture /p/ (Browman and Goldstein, 1990b; Hall, 2010). The AP model accounts for rate-dependent speech sound errors by gestural overlap and gestural magnitude reduction (Browman and Goldstein, 1990b; Hall, 2010). Auditory-perceptual based transcription procedures would describe the schwa elision and consonant deletion (or assimilation processes) in the above examples by a set of phonological rules schematically represented as d →∅/C_C (i.e., /d/ is deleted in the presence of two adjacent consonants in *“nabbed most”* → *“nab most”*; Hall, 2010). However, these rules do not capture the fact that movements for the /d/ or /n/ are still present. Furthermore, articulatory data indicate that such speech sound errors are often not the result of whole-segment or feature substitutions/deletions, but are due to co-production of unintended or intrusion gestures to maintain the dynamic stability in the speech production system instead (Pouplier and Goldstein, 2005; Goldstein et al., 2007; Pouplier, 2007, 2008; Slis and van Lieshout, 2016a,b).

The concept of intrusion gestures is illustrated with kinematic data from Goldstein et al. (2007) study where participants repeated bisyllabic sequences such as “cop top” at fast and slow speech rate conditions. Goldstein et al. (2007) noticed unique speech sound errors in that both the intended and extra/unintended (intruding) gestures were produced at the same time. True substitutions and deletions of the targets occurred rarely, even though, substitution errors are the most commonly reported error type in speech sound error studies when using auditory-perceptual transcription procedures (Dell et al., 2000). Goldstein et al. (2007) explained their findings based on the DST concepts of stable rhythmic synchronization and multi-frequency locking (see Section Gestures, Synergies and Linguistic Contrast). The word pairs “cop top” differ in their onset consonant but share the syllable rhyme. Thus, each production of “cop top” contains one tongue tip (/t/), one tongue dorsum (/k/) gesture, but two labial (/p/) gestures. This results in the initial consonants being in a 1:2 relationship with the coda consonant. Such multi-frequency ratios are intrinsically less stable (Haken et al., 1996), especially under fast rate conditions. As speech rate increased, they observed an extra copy of tongue tip inserted or co-produced during the /k/ production in “cop” and a tongue dorsum intrusion gesture during the /t/ production in “top.” Adding an extra gesture (the intrusion) results in a more stable harmonic relationship where both the initial consonants (tongue tip and tongue dorsum gestures) are in a 2:2 (or 1:1) relationship with the coda (lip gestures) consonant (Pouplier, 2008; Slis and van Lieshout, 2016a,b). Thus, gestural intrusion errors can be described as resulting from a rhythmic synchronization process, where the more complex and less stable 1:2 frequency-locked coordination mode is dissolved and replaced by a simpler and intrinsically more stable 1:1 mode by adding gestures. Unlike what is claimed for perception-based speech sound errors (e.g., Dell et al., 2000), the addition of “extra” cycles of the tongue tip and/or tongue dorsum oscillators results in phonotactically illegal simultaneous articulation of /t/ and /k/ (Goldstein et al., 2007; Pouplier, 2008; van Lieshout and Goldstein, 2008; Slis and van Lieshout, 2016a,b). The fact that /kt/ co-production is phonotactically illegal in English makes it difficult for a listener to even detect its presence. Pouplier and Goldstein (2005) further suggest that listeners only perceive intrusions that are large in magnitude (frequently transcribed as segmental substitutions errors), while smaller gestural intrusions are not heard, and targets are scored as error-free despite conflicting articulatory data (Pouplier and Goldstein, 2005; Goldstein et al., 2007; see also Mowrey and MacKay, 1990).

## Articulatory Phonology and Speech Sound Disorders (SSD) in Children

In this section, we briefly describe the patterns of speech sound errors in children as they have been typically discussed in the S-LP literature. This is followed by an explanation of how the development, maturation, and the combinatorial dynamics of articulatory gestures (such as phasing or timing relationships, coupling strength and gestural overlap) can offer a well-substantiated explanation for several of these more atypical speech sound errors. We will provide a preliminary and arguably, tentative mapping between several subtypes of SSDs in children and their potential origins as explained in the context of the AP and TD framework (Table 1). We see this as a starting point for further discussion and an inspiration to conduct more research in this specific area. For example, one could use the AP/TD model (TADA; Nam et al., 2004) to simulate specific problems at the different levels of the model to systematically probe the emerging symptoms in movement and acoustic characteristics and then verify those with actual data, similar to recent work on apraxia and stuttering using the DIVA framework (Civier et al., 2013; Terband et al., 2019b). Since there is no universally agreed-upon classification system in speech-language pathology, we will limit our discussion to the SSD classification system proposed by Shriberg (2010; Vick et al., 2014; see Waring and Knight, 2013 for a critical evaluation of the current childhood SSD classification systems) and phonological process errors as described in the widely used clinical assessment tool Diagnostic Evaluation of Articulation and Phonology (DEAP; Dodd et al., 2006). We will refer to these phonological error patterns as process errors/speech sound error patterns, in line with their contemporary usage as descriptive terms, without reference to phonological or phonetic theory underpinnings.

**TABLE 1:** Depicts speech sound disorder classification (and subtypes; based on Vick et al., 2014; Shriberg, 2017), most commonly noted error types, examples, and proposed levels of breakdown or impairment within the Articulatory Phonology model and Task Dynamics Framework (Saltzman and Munhall, 1989; Browman and Goldstein, 1992).

Classification or subtype	Error type	Examples	Proposed levels of breakdown
Speech Delay (Process Errors)	Gliding	/ræbIt/ → [wæbIt]	Tract variable, Gestural score
	Vocalization of liquids	/æpl/ → [æpʊ]	Tract variable, Gestural score
	Velar fronting	/go/ → [do]	Tract variable
	Coronal backing	/tu/ → [ku]	
	Palatal fronting (depalatalization)	// →[s]	
	Backing of fricatives	/s/ →/[ʃ]	
	Stopping of fricatives	/zu/ → [du]	Tract variable
	Prevocalic voicing	/pIg/ → [bIg]	Gestural planning oscillators
	Postvocalic devoicing	/bæg/ → [bæk]	
	Weak syllable deletion	/tɛləfoʊn/ → [tɛfoʊn]	Gestural planning oscillators
	Vowel epenthesis	/pliz/ → [pəliz]	Gestural planning oscillators, Inter-gestural coordination.
	Vowel additions	/bæt/ → [bæta]	
	Final consonant deletion	/sit/ → [si]	Gestural planning oscillators, Inter-gestural coordination.
	Cluster reduction	/sneIk/ → [neIk] [seIk]	Inter-gestural coordination, Gestural score Activation.
Articulation Impairment	/s/ and /r/distortions	[sʌn] → [ɬʌn] or [s̪ʌn]	Tract variable
Childhood apraxia of speech (CAS)	(a) Inconsistent speech errors on repeated productions, (b) Lengthened and disrupted coarticulatory transitions between sounds and syllables, and (c) Inappropriate prosody that includes both lexical and phrasal stress difficulties (ASHA, 2007).		Inter-gestural coupling graphs, Inter-gestural planning oscillators, Gestural score activation, Inter-gestural timing, Gesture activation durations, Dynamic gestural specifications at the level of tract variables and articulatory synergies.
Speech Motor Delay (SMD)	(a) Immature motor control system. (b) Higher articulatory kinematic variability of upper lip, lower lip and jaw, larger upper lip displacements. (c) Fewer accurate phonemes, errors in vowel and syllable duration, errors in glide production, epenthesis errors, consonantal distortions, and less accurate lexical stress.		Inter-gestural planning oscillators Gestural score activation Inter-gestural timing Gesture activation durations Dynamic gestural specifications at the level of tract variables and articulatory synergies
Developmental dysarthria	(a) Neuro-motor timing and execution (b) Reduced speaking rates and prolonged syllable durations. (c) Decreased vowel distinctiveness and sound distortions, (d) Reduced strength of articulatory contacts (e) Voice and prosodic abnormalities (f) Reduced respiratory support and/or incoordination		Inter-gestural coordination and dynamic specifications at the level of Tract variables and Articulatory Synergies

### Speech Delay

According to Shriberg et al. (2010) and Shriberg et al. (2017), children with Speech Delay (age of occurrence between 3 and 9 years) are characterized by “delayed acquisition of correct auditory–perceptual or somatosensory features of underlying representations and/or delayed development of the feedback processes required to fine tune the precision and stability of segmental and suprasegmental production to ambient adult models” (Shriberg et al., 2017, p. 7). These children present with age-inappropriate speech sound deletions and/or substitutions, among which patterns of speech sound errors as described below:

#### Gliding and Vocalization of Liquids

Gliding is described as a substitution of a liquid with a glide (e.g., rabbit /ræbIt/ → [wæbIt] or [jæbIt], please /pliz/ → [pwiz], look /lʊk/ → [wʊk]; McLeod and Baker, 2017) and vocalization of liquids refers to the substitution of a vowel sound for a liquid (e.g., apple /æpl/ → [æpʊ], bottle /bɑtl/ → [bɑtʊ]; McLeod and Baker, 2017). The /r/ sounds are acoustically characterized by a drop in the third formant (Alwan et al., 1997). In terms of movement kinematics the /r/ sound is a complex coproduction of three vocal tract constrictions/gestures (i.e., labial, tongue tip/body, and tongue root), requires a great deal of speech motor skill, and is mastered by most typically developing children between 4 and 7 years of age (Bauman-Waengler, 2016). Ultrasound data suggests that children may find the simultaneous coordination of three gestures motorically difficult and may simplify the /r/ production by dropping one gesture from the segment (Adler-Bock et al., 2007). Moreover, the syllable final /r/ sounds are often substituted with vowels because they share only a subset of vocal tract constrictions with the original /r/ sound and this is better described as a simplification process (Adler-Bock et al., 2007). For example, the child may drop the tongue tip gesture but retain the lip rounding gesture and the latter dominates resulting vocal tract acoustics (Adler-Bock et al., 2007; van Lieshout et al., 2008). Kinematic data derived from electromagnetic articulography (van Lieshout et al., 2008) also points to a limited within-organ differentiation of the tongue parts and subtle issues in relative timing between different components of the tongue in /r/ production errors. These arguments also have support from longitudinal observational data on positional lateral gliding in children (/l/ is realized as [j]; Inkelas and Rose, 2007). Positional lateral gliding in children is said to occur when the greater gestural magnitude of prosodically strong onsets in English interacts with the anatomy of the child’s vocal tract (Inkelas and Rose, 2007; McAllister Byun, 2011, 2012). Within the AP model, reducing the number of required gestures (simplification) and poor tongue differentiation issues would likely have their origins at the level of *Tract Variables* while issues in relative timing between the tongue gestures are likely to arise at the level of the *Gestural Score* (Table 1).

#### Stopping of Fricatives

Stopping of fricatives involves a substitution of a fricative consonant with a homorganic plosive (e.g., zoo /zu/ → [du], shoe /ʃu/ → [tu], see /si/ → [ti]; McLeod and Baker, 2017). Fricatives are another class of late acquired sounds that require precise control over different parts of the tongue to produce a narrow groove through which turbulent airflow passes. Within the AP model, the stopping of fricatives may arise from an inappropriate *Tract Variable* constriction degree specification (Constriction Degree: /d/ closed vs. /z/ critical; Goldstein et al., 2006; see Table 1), possibly as a simplification process secondary to limited precision of tongue tip control. Alternatively, neutralization (or stopping) of fricatives especially in prosodically strong contexts has also been explained from a constraint-based grammar perspective. For example, the tendency to overshoot is greater in initial positions where a more forceful gesture is favored for prosodic reasons. This allows the hard to produce fricative to be replaced by a ballistic tongue-jaw gesture that does not violate the MOVE-AS-UNIT constraint (Inkelas and Rose, 2007; McAllister Byun, 2011, 2012) as described in the “Introduction Section.”

#### Vowel Addition and Final Consonant Deletion

Different types of vowel insertion errors have been observed in children’s speech. An epenthesis is typically a schwa vowel inserted between two consonants in a consonant cluster (e.g., please /pliz/ → [pəliz] CCVC → CVCVC; blue /blu/ → [bəlu] CCV → CVCV), while other types of vowel insertions have also been noted (e.g., bat /bæt/ → [bæta]; CVC → CVCV) (McLeod and Baker, 2017). A final consonant deletion involves the deletion of a consonant in a syllable or word final position (seat /sit/ → [si], cat /cæt/ → [cæ], look /lʊk/ → [lʊ]; McLeod and Baker, 2017). Both these phenomena could be explained by the concept of relative stability. As noted earlier, the onset consonant and the vowel (CV) are coupled in a relatively more stable in-phase mode as opposed to the anti-phase VC and CC gestures (Goldstein et al., 2006; Nam et al., 2009; Giulivi et al., 2011). Thus, the maintenance of relative stability in VC or CC coupling modes may be more difficult with increasing cognitive-linguistic (e.g., vocabulary growth) or speech motor demands (e.g., speech rate), and there may be a tendency to utilize intrusion gestures as a means to stabilize the speech motor system (i.e., by decreasing frequency locking ratios; e.g., 2:1 to 1:1; Goldstein et al., 2007). We suspect that such mechanisms underlie vowel intrusion (error) gestures in children. In CVC syllables (or word structures), greater stability in the system may be achieved by dropping or deleting the final consonant and thus retaining the more stable in-phase CV coupling (Goldstein et al., 2006). Moreover, findings from ultrasound tongue motion data during the production of repeated two- and three-word phrases with shared consonants in coda (e.g., top cop) versus no-coda positions (e.g., taa kaa, taa kaa taa) have demonstrated a gestural intrusion bias only for the shared coda consonant condition (Pouplier, 2008). These findings suggest that the presence of (shared) coda consonants is a trigger for a destabilizing influence on the speech motor system (Pouplier, 2008; Mooshammer et al., 2018). From an AP perspective, the stability induced by deleting final consonants or adding intrusion gestures (lowering frequency locking ratios) can be assigned to limitations in inter-gestural coordination and/or possible gestural selection issues at the level of *Gestural Planning Oscillators* (Figure 2). We argue that (vowel) intrusion sound errors are not a “symptom” of an underlying (phonological) disorder, but rather the result of a compensatory mechanism for a less stable speech motor system. Additionally, children with limited jaw control may omit the final consonant /b/ in /bɑb/ in a jaw close-open-close production task, due to difficulties with elevating the jaw. This would typically be associated with the *Tract Variable* level in the AP model or at later stages during the specification of jaw movements at the *Articulatory* level (see Figure 2 and Table 1).

#### Cluster Reduction

Cluster reduction refers to the deletion of a (generally more marked) consonant in a cluster (e.g., please /pliz/ → [piz], blue /blu/ → [bu], spot /spɒt/ → [pɒt]; McLeod and Baker, 2017). From a stability perspective, CC onset clusters are less stable (i.e., anti-phasic) and in the presence of increased demands or limitations in the speech motor system (e.g., immaturity; Fletcher, 1992), they are more likely replaced by a stable CV coupling pattern by omitting the extra consonantal gesture (Goldstein et al., 2006; van Lieshout and Goldstein, 2008; Nam et al., 2009). Alternatively, there is also the possibility that when two (heterorganic) gestures in a cluster are produced they may temporally overlap, thereby acoustically and perceptually hiding one gesture (i.e., gestural hiding; Browman and Goldstein, 1990b; Hardcastle et al., 1991; Gibbon et al., 1995). Within the AP model, cluster reductions due to stability factors and gestural hiding may be ascribed to the *Gestural Score Activation* level (a gesture may not be activated in a CCV syllable to maintain stable CV structure) and to relative phasing issues (increased temporal overlap) at the level of inter-gestural coordination (Figure 2 and Table 1; Goldstein et al., 2006; Nam et al., 2009).

#### Weak Syllable Deletion

Weak syllable deletion refers to the deletion of an unstressed syllable (e.g., telephone /tɛləfoʊn/ → [tɛfoʊn], potato /pəteɪtoʊ/ → [teɪtoʊ], banana /bənænə/ → [nænə]; McLeod and Baker, 2017). Multisyllabic words pose a unique challenge in that they comprise of complex couplings between multi-frequency syllable and stress level oscillators (e.g., Tilsen, 2009). Deleting an unstressed syllable in a multisyllabic word may allow reduction of complexity by frequency locking in a stable lower order-mode between syllable and stress level oscillators. Within the AP model, this process is regulated at the level of *Gestural Planning Oscillators* (see Table 1; Goldstein et al., 2007; Tilsen, 2009).

#### Velar Fronting and Coronal Backing

Fronting is defined as a substitution of a sound produced in the back of the vocal tract with a consonant articulated further toward the front (e.g., go /go/ → [do], duck /dk/ → [dt], key /ki/ → [ti]; McLeod and Baker, 2017). Backing on the other hand, is defined as a substitution of a sound produced in the front of the vocal tract with a consonant articulated further toward the back (e.g., two /tu/ → [ku], pat /pæt/ → [pæk], tan /tæn/ → [kæn]; McLeod and Baker, 2017). While fronting is frequently observed in typically developing young children, backing is rare for English-speaking children (McLeod and Baker, 2017). Children who exhibit fronting and backing behaviors show evidence of undifferentiated lingual gestures, according to electropalatography (EPG) and electromagnetic articulography studies (Gibbon, 1999; Gibbon and Wood, 2002; Goozée et al., 2007). Undifferentiated lingual gestures lack clear differentiation between the movements of the tongue tip, tongue body, and lateral margins of the tongue. For example, tongue-palate contact is not confined to the anterior part of the palate for alveolar targets, as in normal production. Instead, tongue-palate contact extends further back into the palatal and velar regions of the vocal tract (Gibbon, 1999). It is estimated that 71% of children (aged 4-12 years) with a clinical diagnosis of articulation and phonological disorders produce undifferentiated lingual gestures. These undifferentiated lingual gestures are argued to arise from decreased oro-motor control abilities, a deviant compensatory bracing mechanism (i.e., an attempt to counteract potential disturbances in tongue tip fine motor control; Goozée et al., 2007) or may represent an immature speech motor system (Gibbon, 1999; Goozée et al., 2007). Undifferentiated lingual gestures are not a characteristic of speech in typically developing older school-age children or adults (Gibbon, 1999). In children’s productions of lingual consonants, there is a decrease in tongue-palate contact on EPG with increasing age (6 through 14 years) paralleled by fine-grained articulatory adjustments (Fletcher, 1989). The tongue tip and tongue body function as two quasi-independent articulators in typical and mature speech production systems (see section *Development of Synergies in Speech*). However, in young children, the tongue and jaw (tongue-jaw complex) and different functional parts of the tongue may be strongly coupled in-phase (i.e., always move together), and thus lack functionally independent regions (Gibbon, 1999; Green et al., 2002). Undifferentiated lingual patterns may thus result from simultaneous (in-phase) activation of regions of the tongue and/or tongue-jaw complex in young children and persist over time (van Lieshout et al., 2008).

Standard acoustic-perceptual transcription procedures do not reliably detect undifferentiated lingual gestures (Gibbon, 1999). Undifferentiated lingual gestures are sometimes transcribed as phonetic distortions or phonological substitutions (i.e., velar fronting or coronal backing) in some contexts, but may be transcribed as correct productions in other contexts (Gibbon, 1999; Gibbon and Wood, 2002). The perception of place of articulation of an undifferentiated gesture is determined by changes in tongue-palate contact during closure (i.e., *articulatory drift;* Gibbon and Wood, 2002). For example, closure might be initiated in the velar region, cover the entire palate, and then be released in the coronal or anterior region (or vice versa). Undifferentiated lingual gestures could therefore yield the perception of either velar fronting or coronal backing. The perceived place of articulation is influenced by the direction of the articulatory drift and the last tongue-palate contact region (Gibbon and Wood, 2002). Children with slightly more advanced lingual control, relative to those described with widespread use of undifferentiated gestures, may still present with fine-motor control or refinement issues (e.g., palatal fronting /ʃ/ →[s]; backing of fricatives /s/ →[ʃ]; Gibbon, 1999). Velar fronting and coronal backing can be envisioned as incorrect in relative phasing at the level of inter-gestural coordination^3^ (see Table 1). For instance, the tongue tip-tongue body or tongue-jaw complex may be in a tight synchronous in-phase coupling, but the release of constriction may not. It may also be a problem in *Tract Variable* constriction location specification (Table 1).

#### Prevocalic Voicing and Postvocalic Devoicing

Context sensitive voicing errors in children are categorized as prevocalic voicing and postvocalic devoicing. *Prevocalic voicing* is a process in which voiceless consonants in syllable initial positions are replaced by voiced counterparts (e.g., pea /pi/ → [bi]; pan /pæn/ → [bæn]; pencil /pεnsəl/ → [bεnsəl]) and *postvocalic devoicing* is when voiced consonants in syllable final position are replaced by voiceless counterparts (e.g., Bag /bæg/ → [bæk], pig /pIg/ → [pIk]; seed /sid/ → [sit]; McLeod and Baker, 2017). Empirical evidence suggests that in multi-gestural segments, segment-internal coordination of gestures may be different in onset than in coda position (Krakow, 1993; Goldstein et al., 2006). When a multi-gestural segment is produced in a syllable onset, such as a bilabial nasal stop (e.g., [m]), the necessary gestures (bilabial closure gesture, glottal gesture and velar gesture) are synchronously produced (i.e., in-phase), creating the most stable configuration for that combination of gesture; this makes the addition of voicing in onset position easy. However, in coda position, the bilabial closure gesture, glottal gesture (for voicing) and velar gesture must be produced asynchronously (i.e., in a less stable anti-phase mode; Haken et al., 1985; Goldstein et al., 2006, 2007). It is thus less demanding to coordinate fewer gestures in the anti-phase mode across oral and laryngeal speech subsystems in a coda position. This would explain why children (with a developing speech motor system) may simply drop the glottal gesture (devoicing in coda position) to reduce complexity. Note, that in some languages (e.g., Dutch), coda devoicing is standard irrespective of the original voicing characteristic of that sound. Within the AP model, prevocalic voicing and postvocalic devoicing (i.e., adding or dropping a gesture) may be ascribed to gestural selection issues at the level of *Gestural Planning Oscillators* (Figure 2 and Table 1).

Recent studies also suggest a relationship between jaw control and acquisition of accurate voice-voiceless contrasts in children. The production of a voice-voiceless contrast requires precise timing between glottal abduction/adduction and oral closure gestures. Voicing contrast acquisition in typically developing 1- to 2-year-old children may be facilitated by increasing the jaw movement excursion, speed and stability (Grigos et al., 2005). In children with SSDs (including phonological disorder, articulation disorder and CAS) relative to typically developing children, jaw deviances/instability in the coronal plane (i.e., lateral jaw slide) have been observed (Namasivayam et al., 2013; Terband et al., 2013). Moreover, stabilization of voice onset times for /p/ production has been noted in children with SSDs undergoing motor speech intervention focused on jaw stabilization (Yu et al., 2014). These findings are not surprising given that the perioral (lip) area lacks tendon organs, joint receptors and muscle spindles (van Lieshout, 2015), and the only reliable source of information to facilitate inter-gestural coordination between oral and laryngeal gestures comes from the jaw masseter muscle spindle activity (Namasivayam et al., 2009). Increases in jaw stability and amplitude may provide consistent and reliable feedback used to stabilize the output of a coupled neural oscillatory system comprising of larynx (glottal gestures) and oral articulators (van Lieshout, 2004; Namasivayam et al., 2009; Yu et al., 2014; van Lieshout, 2017).

### Articulation Impairment

Articulation impairment is considered a motor speech difficulty and generally reserved for speech sound errors related to rhotics and sibilants (e.g., derhotacized /r/: bird /bɝd/ → [bɜd]; dentalized/lateralized sibilants: sun /sn/ → [ɬʌn] or [s̪ʌn]; McLeod and Baker, 2017). A child with an articulation impairment is assumed to have the correct phoneme selection but is imprecise in the speech motor specifications and implementation of the sound (Preston et al., 2013; McLeod and Baker, 2017). Studies using ultrasound, EPG and electromagnetic articulography data have shown several aberrant motor patterns to underlie sibilant and rhotic distortions. For rhotics, these may range from undifferentiated tongue protrusion, absent anterior tongue elevation, absent tongue root retraction and subtle issues in relative timing between different components of the tongue gestures (van Lieshout et al., 2008; Preston et al., 2017). Correct /s/ productions involve a groove in the middle of the tongue along with an elevation of the lateral tongue margins (Preston et al., 2016, 2017). Distortions in /s/ production may arise from inadequate anterior tongue control, poor lateral bracing (sides of the tongue down) and missing central groove (McAuliffe and Cornwell, 2008; Preston et al., 2016, 2017).

Within the AP model, articulation impairments may potentially arise at three levels: *Tract Variables*, *Gestural Scores* and dynamical specification of the gestures. We discussed rhotic production issues at the *Tract Variables* and *Gestural Score* levels in the *Gliding and vocalization of liquids* section as a reduction in the number of required gestures (i.e., some parts of the tongue not activated during /r/), limited tongue differentiation, and/or subtle relative timing issues between the different tongue gestures/components. Errors in dynamical specifications of the gestures could also result in speech sound errors. For example, incorrect damping parameter specification for vocal tract constriction degree may result in the *Tract Variables* (and their associated articulators) overshooting (underdamping) or undershooting (overdamping) their rest/target value (Browman and Goldstein, 1990a; Fuchs et al., 2006).

### Childhood Apraxia of Speech (CAS)

The etiology for CAS is unknown, but it is hypothesized to be a neurological sensorimotor disorder with a disruption at the level of speech motor planning and/or motor programing of speech movement sequences (American Speech–Language–Hearing Association (ASHA, 2007). A position paper by ASHA (2007) describes three important characteristics of CAS which include inconsistent speech sound errors on repeated productions, lengthened and disrupted coarticulatory transitions between sounds and syllables, and inappropriate prosody that includes both lexical and phrasal stress difficulties (ASHA, 2007). Within the AP and TD framework, the speech motor planning processes described in linguistic models can be ascribed to the level of inter-gestural coupling graphs, inter-gestural planning oscillators and gestural score activation; while processes pertaining to speech motor programing would typically encompass dynamic gestural specifications at the level of tract variables and articulatory synergies (Nam and Saltzman, 2003; Nam et al., 2009; Tilsen, 2009).

Traditionally, perceptual inconsistency in speech production of children with CAS has been evaluated via word-level token-to-token variability or at the fine-grained segmental-level (phonemic and phonetic variability; Iuzzini and Forrest, 2010; Iuzzini-Seigel et al., 2017). These studies provide evidence for increased variability in speech production of CAS relative to those typically developing or those with other speech impairments (e.g., articulation disorders). Data suggest that speech variability issues in CAS may arise at the level of articulatory synergies (intra-gestural coordination). Children with CAS demonstrate higher lip-jaw spatio-temporal variability with increasing utterance complexity (e.g., word length: mono-, bi-, and tri-syllabic) and greater lip aperture variability relative to children with speech delay (Grigos et al., 2015). Terband et al. (2011) analyzed articulatory kinematic data on functional synergies in 6- to 9-year-old children with SSD, CAS, and typically developing controls. The results indicated that the tongue tip-jaw synergy was less stable in children with CAS compared to typically developing children, but the stability of lower lip-jaw synergy did not differ (Terband et al., 2011). Interestingly, differences in movement amplitude emerged between the groups: CAS children exhibited a larger contribution of the lower lip to the oral closure compared to typically developing controls, while the children with SSD demonstrated larger amplitude of tongue tip movements relative to CAS and controls. Terband et al. (2011) suggest that children with CAS may have difficulties in the control of both lower lip and tongue tip while the children with SSD have difficulties controlling only the tongue tip. Larger movement amplitudes found in these groups may indicate an adaptive strategy to create relatively stable movement coordination (see also Namasivayam and van Lieshout, 2011; van Lieshout, 2017). The presence of larger movement amplitudes to increase stability in the speech motor system has been reported as a potential strategy in other speech disorders, including stuttering (Namasivayam et al., 2009); adult verbal apraxia and aphasia (van Lieshout et al., 2007); cerebral palsy (Nip, 2017; Nip et al., 2017); and Speech-Motor Delay [SMD, a SSD subtype formerly referred to as Motor Speech Disorder–Not Otherwise Specified (MSD-NOS); Vick et al., 2014; Shriberg, 2017; Shriberg et al., 2019a,b]. This fits well with the notion that movement amplitude is a factor in the stability of articulatory synergies as predicted in a DST framework (e.g., Haken et al., 1985; Peper and Beek, 1998) and evidenced in a recent study on speech production (van Lieshout, 2017). Additional mechanisms to improve stability in movement coordination were documented in gestural intrusion error studies (Goldstein et al., 2007; Pouplier, 2007, 2008; Slis and van Lieshout, 2016a,b) as discussed in section “Describing Casual Speech Alternations,” and are more present in adult apraxia speakers relative to healthy controls (Pouplier and Hardcastle, 2005; Hagedorn et al., 2017).

With regards to the lengthened and disrupted coarticulatory transitions, findings suggest that abnormal and variable anticipatory coarticulation (assumed to reflect speech motor planning) may be specific to CAS and not a general characteristic of children with SSD (Nijland et al., 2002; Maas and Mailend, 2017). The lengthened and disrupted coarticulatory transitions between sounds and syllables can be explained by possible limitations in inter-gestural overlap in children with CAS. A reduction in overlap of successive articulatory gestures (i.e., reduced coarticulation or coproduction) may result in the speech output becoming “segmentalized” (e.g., as seen in adult apraxic speakers; Liss and Weismer, 1992). *Segmentalization* gives the perception of “pulling apart” of successive gestures in the time domain and possibly adds to perceived stress and prosody difficulties in this population (e.g., Weismer et al., 1995). These may arise from delays in the activation of the following gesture and/or errors in gesture activation durations.

Inappropriate prosody (lexical and phrasal stress difficulties) in CAS is often characterized by listener perceptions of misplaced or equalized stress patterns across syllables. A potential source of this problem is that children with CAS may produce subtle and not consistently perceptible acoustic differences between stressed and unstressed syllables (Shriberg et al., 1997; Munson et al., 2003). Children with CAS unlike typically developing children, do not shorten vowel duration in weaker stressed initial syllables as an adjustment to the metrical structure of the following syllable (Nijland et al., 2003). Furthermore, syllable omissions have been particularly noted in CAS children who demonstrated inappropriate phrasal stress (Velleman and Shriberg, 1999). These interactions between syllable/gestural units and rhythmic (stress and prosody) systems have been discussed earlier in the context of multi-frequency systems of coupled oscillators (e.g., Tilsen, 2009). We speculate that children with CAS may have difficulty with stability in coupling (i.e., experience weak or variable coupling) between stress and syllable level oscillators.

### Speech-Motor Delay

Speech-Motor Delay (formerly MSD-NOS; Vick et al., 2014; Shriberg, 2017; Shriberg and Wren, 2019; Shriberg et al., 2019a,b) is a subpopulation of children presenting with difficulties in speech motor control and coordination that is not consistent with features of CAS or Dysarthria (Shriberg, 2017; Shriberg et al., 2019a,b). Information on the nature, diagnosis, and intervention protocols for the SMD subpopulation is emerging (Vick et al., 2014; Shriberg, 2017; Namasivayam et al., 2019). Current data suggests that this group is characterized by poor motor control (e.g., higher articulatory kinematic variability of upper lip, lower lip and jaw, larger upper lip displacements). Behaviorally, they produce errors such as fewer accurate phonemes, errors in vowel and syllable duration, errors in glide production, epenthesis errors, consonantal distortions, and less accurate lexical stress (Vick et al., 2014; Shriberg, 2017; Namasivayam et al., 2019; Shriberg and Wren, 2019; Shriberg et al., 2019a,b). As many of the precision and stability deficits in speech and prosody in SMD (e.g., consonant distortions, epenthesis, vowel duration differences and decreased accuracy of lexical stress) and adaptive strategies to increase speech motor stability (e.g., larger upper lip displacements; van Lieshout et al., 2004; Namasivayam and van Lieshout, 2011) overlap with CAS and other disorders discussed earlier, we will not reiterate possible explanations for these within the context of the AP model. SMD is considered a disorder of execution: a delay in the development of neuromotor precision-stability of speech motor control. Children with SMD are at increased risk for persistent SSDs (Shriberg et al., 2011, 2019a,b; Shriberg, 2017).

### Developmental Dysarthria

Dysarthria “is a collective name for a group of speech disorders resulting from disturbances in muscular control over the speech mechanism due to damage of the central or peripheral nervous system. It designates problems in oral communication due to paralysis, weakness, or incoordination of the speech musculature” (Darley et al., 1969, p. 246). Dysarthria may be present in children with cerebral palsy (CP) and may be characterized by reduced speaking rates, prolonged syllable durations, decreased vowel distinctiveness, sound distortions, reduced strength of articulatory contacts, voice abnormalities, prosodic disturbances (e.g., equal stress), reduced respiratory support or respiratory incoordination and poor intelligibility (Pennington, 2012; Mabie and Shriberg, 2017; Nip et al., 2017). Speakers with CP consistently produce greater lip, jaw and tongue displacements in speech tasks relative to typically developing peers (Ward et al., 2013; Nip, 2017; Nip et al., 2017). These increased displacements were argued to arise from either a reduced ability to grade force control (resulting in ballistic movements) or alternatively, can be interpreted as a strategy to increase proprioceptive feedback to stabilize speech movement coordination (Namasivayam et al., 2009; Nip, 2017; Nip et al., 2017; van Lieshout, 2017). Further, children with CP demonstrate decreased spatial coupling between the upper and lower lips and reduced temporal coordination between the lips and between lower lip and jaw (Nip, 2017) relative to typically developing peers. These measures of inter-articulator coordination were found to be significantly correlated with speech intelligibility (Nip, 2017).

Within the AP model, the neuromotor characteristics of dysarthria such as disturbances in gesture magnitude or scaling issues (overshooting, undershooting), imprecise articulatory contacts (resulting in sound distortions), slowness (reduced speaking rate and prolonged durations), and coordination issues could be related to inaccurate gestural specifications of dynamical parameters (e.g., damping and stiffness), inaccurate gesture activation durations, imprecise constriction location and degree, and inter-gestural and intra-gestural (i.e., articulatory synergy level) timing issues (Browman and Goldstein, 1990a; van Lieshout, 2004; Fuchs et al., 2006). Inter-gestural and intra-gestural timing issues may characterize difficulties in coordinating the subsystems required for speech production (respiration, phonation and articulation) and difficulties in controlling the many degrees of freedom in a functional articulatory synergy, respectively (Saltzman and Munhall, 1989; Browman and Goldstein, 1990b; van Lieshout, 2004). Overall, dysarthric speech characteristics would encompass the following levels in the AP/TD framework: inter-gestural coordination, and dynamic specifications at the level of *Tract Variables* and *Articulatory Synergies* (Table 1).

## Clinical Relevance, Limitations and Future Directions

In this paper, we briefly reviewed some of the key concepts from the AP model (Browman and Goldstein, 1992; Gafos and Goldstein, 2012). We explained how the development, maturation, and the combinatorial dynamics of articulatory gestures in this model can offer plausible explanations for speech sound errors found in children with SSDs. We find that many of these speech sound error patterns are in fact present in speech of typically developing children and more importantly, even in the speech of typical adult speakers, under certain circumstances. Based on our presentation of behavioral and articulatory kinematic data we propose that such speech sound errors in children with SSD may potentially arise as a consequence of the complex interaction between the dynamics of articulatory gestures, an immature speech motor system with limitations in speech motor skills and specific boundary conditions related to physical, physiological, and functional constraints. In fact, much of these speech sound errors themselves may reflect compensatory strategies (e.g., decreasing speech rate, increasing movement amplitude, bracing, intrusion gestures, cluster reductions, segment/gesture/syllable deletions, increasing lag between articulators) to provide more stability in the speech motor system as has been found in both typical and disordered speakers (Fletcher, 1992; van Lieshout et al., 2004; Namasivayam and van Lieshout, 2011).

Based on the presented evidence, we speculate that in general children with SSDs may occupy the low end of the speech motor skill continuum similar to what has been argued for stuttering (van Lieshout et al., 2004; Namasivayam and van Lieshout, 2011) and that the differences we notice in speech sound errors between the subtypes of SSD may in fact be differences in how these individuals develop strategies for coping with the challenges of being on the lower end of the speech motor skill continuum. This is a critical shift in thinking about the (distal and proximal) causes for speech sound errors in children with SSD (or in adults for that matter). Many of these children show similarities in their behavioral symptoms and perhaps the traditional notion of separating phonological from motor issues should be questioned (see also Maassen et al., 2010) and replaced with a broader understanding of how all levels involved in speech production are part of a complex system with processing stages that are highly integrated and coupled at different time scales (see also Tilsen, 2009, 2017). The AP perspective and the associated DST principles provide a suitable basis for this kind of approach given its transparency between higher and lower levels of control through the concept of gestures.

Despite the uniqueness of the AP approach in offering new insights into the underlying mechanisms of speech sound errors in children, there are some limitations of using this approach. For example, the current versions of the AP model does not have an auditory feedback channel and is unable to account for any effects of auditory feedback perturbations. Further, although there are some recent attempts at describing the neural mechanisms underlying the components of the AP model (e.g., Tilsen, 2016) the model generally does not explicitly specify neural structures as some other models have done (e.g., DIVA model; Tourville and Guenther, 2011; for a detailed comparison between models of speech production see Parrell et al., 2019).

Critically, the theoretical concepts of gestures/synergies in speech production from this framework are yet to be taught widely in professional S-LP programs and related disciplines (see also van Lieshout, 2004). There are several reasons for this knowledge translation issue with the top ones being a lack of availability of accessible reviews and tutorials on this topic, limited empirical data on the nature of SSDs in children from an AP framework, and most importantly the absence of convenient, reliable and published practical methods to assess the status of gestures and synergies in speech production in a clinical setting. Although, some intervention approaches like the Prompts for Restructuring Oral Muscular Phonetic Targets approach (PROMPT; Hayden et al., 2010) and the Rapid Syllable Transitions Treatment program (ReST; Thomas et al., 2014) aim at addressing speech movement gestures and transitions between them, they lack empirical outcome data related to their impact at the level of gestures and articulatory synergies. It is also unclear at this point whether or not it is possible to provide tools to identify differences in timing relationships in jaw-lip or tongue tip-jaw coupling that would work well in a clinical setting. Using purely sensory (visual and auditory) means to observe speech behaviors will always be subject to errors and biases common to perception-based evaluation procedures (e.g., Kent, 1996). At the moment, there is a paucity of literature in this area which opens up great opportunities for future research. With technologies like real time Magnetic Resonance Imaging finding its way into the analysis of typical and disordered speech (e.g., see Hagedorn et al., 2017) and relatively low cost automatic video-based face-tracking systems (Bandini et al., 2017) starting to emerge for clinical purposes, we hope that speech-language pathologists will have the tools they need to support their assessment and intervention planning based on a better understanding and quantification of the dynamics of speech gestures and articulatory synergies. To this end, we hope that this paper provides an initial step in this direction as an introduction to the AP framework for clinical audiences and a motivation for a larger cohort of researchers for developing testable hypothesis regarding the contribution of gestures and articulatory synergies to sub-types of SSD in children.

## Conclusion

The foundations of clinical assessment, classification and intervention for children with SSD have been heavily influenced by psycholinguistics and auditory-perceptual based transcription procedures (Shriberg, 2010; see Section *Articulatory Phonology and Speech Sound Disorders in Children*). A major problem as noted earlier (in the Introduction section) is that, the complex relationships between the etiology (distal), processing deficits (proximal) and the behavioral levels (speech symptoms) is under-specified in current SSD classification systems (Terband et al., 2019a). It is critical to understand the complex interactions between these levels as they have implications for differential diagnosis and treatment planning (Terband et al., 2019a). There have been some theoretical attempts made toward understanding these interactions (e.g., Inkelas and Rose, 2007; McAllister Byun, 2012; McAllister Byun and Tessier, 2016), and we hope this paper will trigger a stronger interest in the field of S-LP for an alternative “gestural” perspective and increase the contributions to the limited corpus of research literature in this area.

## Author Contributions

AN: main manuscript writing, synthesis and interpretation of literature, brain storming concepts and ideas, and creation of tables and figures. DC and AO: main manuscript writing, brain storming concepts and ideas, references, and proofing. PL: overall supervision of manuscript, writing subsections, and original conceptualization.

## Conflict of Interest

The authors declare that the research was conducted in the absence of any commercial or financial relationships that could be construed as a potential conflict of interest.

## Footnotes

1 The term “speech sound error” refers to a mismatch between what an individual intends to say and what they actually say (Harley, 2006). In children, this may entail a clinically significant impairment or a non-standard production of speech sounds of the ambient language and may be classified according to the units of processing (e.g., phoneme, syllable, word or phrase) and the mechanisms (substitutions, additions, omissions/deletions and distortions) involved (Harley, 2006; Preston et al., 2013). The word “sound” is included in the term “speech sound error” to distinguish it from other speech errors such as disfluencies, voice and language (e.g., grammatical errors) based errors (McLeod and Baker, 2017).

2 There are also certain states that are inherently unstable, which are referred to as repellors.

3 For an alternative take on velar fronting using the Harmonic Grammar framework and AP model see McAllister Byun, 2011, 2012; McAllister Byun and Tessier, 2016.
